# The lateral intraparietal sulcus takes viewpoint changes into account during memory-guided attention in natural scenes

**DOI:** 10.1007/s00429-021-02221-y

**Published:** 2021-02-03

**Authors:** Ilenia Salsano, Valerio Santangelo, Emiliano Macaluso

**Affiliations:** 1 Neuroimaging Laboratory, IRCCS Santa Lucia Foundation, Rome, Italy; 2 PhD Program in Behavioral Neuroscience, Sapienza University of Rome, Rome, Italy; 3 Department of Philosophy, Social Sciences and Education, University of Perugia, Perugia, Italy; 4 ImpAct Team, Lyon Neuroscience Research Center, Lyon, France

**Keywords:** fMRI, Attention, Long-term memory, Intraparietal sulcus, Natural scenes, Visual search

## Abstract

Previous studies demonstrated that long-term memory related to object-position in natural scenes guides visuo-spatial attention during subsequent search. Memory-guided attention has been associated with the activation of memory regions (the medial-temporal cortex) and with the fronto-parietal attention network. Notably, these circuits represent external locations with different frames of reference: egocentric (i.e., eyes/head-centered) in the dorsal attention network vs. allocentric (i.e., world/scene-centered) in the medial temporal cortex. Here we used behavioral measures and fMRI to assess the contribution of egocentric and allocentric spatial information during memory-guided attention. At encoding, participants were presented with real-world scenes and asked to search for and memorize the location of a high-contrast target superimposed in half of the scenes. At retrieval, participants viewed again the same scenes, now all including a low-contrast target. In scenes that included the target at encoding, the target was presented at the same scene-location. Critically, scenes were now shown either from the same or different viewpoint compared with encoding. This resulted in a memory-by-view design (target seen/unseen *x* same/different view), which allowed us teasing apart the role of allocentric vs. egocentric signals during memory-guided attention. Retrieval-related results showed greater search-accuracy for seen than unseen targets, both in the same and different views, indicating that memory contributes to visual search notwithstanding perspective changes. This view-change independent effect was associated with the activation of the left lateral intra-parietal sulcus. Our results demonstrate that this parietal region mediates memory-guided attention by taking into account allocentric/scene-centered information about the objects' position in the external world.

## Introduction

Visuo-spatial attention plays a key role in our everyday experience, allowing us to select relevant information to pursue current behavioral goals. Many different types of signals contribute to the control of spatial attention. Traditional studies with simple and stereotyped stimuli (e.g., geometrical shapes) focused—on one hand—on exogenous signals that relate to the physical characteristics of the stimuli (e.g., capture of attention by a red square presented among green items) and—on the other hand—on task-related endogenous processes. The latter comprise, for example, spatial orienting following informative cues (cf. Posner 1980), reward cues (see, e.g., Maunsell 2004; Gottlieb et al. 2014), or the use of probabilistic information related to trial history (Chun and Jiang 1998; Jiang and Swallow 2014; Preston and Eichenbaum 2013).

Most recently, researchers have started investigating mechanisms of attention control using more complex and realistic stimuli, such as pictures of natural scenes (Summerfield et al. 2006, 2011). In this framework, it has become apparent that long-term knowledge about the layout of objects in natural environments also plays a role in guiding spatial attention (Rosen et al. 2015, 2016; Summerfield et al. 2006; see also Hutchinson and Turk-Browne 2012). Long-term memory (LTM) can provide us with predictive information about the likely location of objects, people, or animals in natural environments, resulting in the optimization of resources allocation, perception and—ultimately—behavior (Chun and Turk-Browne 2007; Hollingworth et al. 2001; Hollingworth 2004; Olivers 2011; Patai et al. 2012; Summerfield et al. 2006, 2011; see Wolfe and Horowitz 2017, for a recent review).

In a pioneering study, Summerfield and colleagues (2006) utilized fMRI to investigate the neural substrates of attention guidance by memory, using pictures of real-world scenes. On the first day, participants were presented with pictures of natural scenes, some of which included a small target-key superimposed on the scene. The task was to report the left/right placement of the key, or its absence. After 1 or 2 days, during fMRI scanning, the participants were asked to detect the target-keys again superimposed over the natural scenes. The target-key was flashed 500–900 ms after the onset of the picture, allowing the participant to orient spatial attention before the target presentation. In different fMRI runs, participants used either their previous knowledge of the target-location (“memory-orienting task”, including old scenes from day 1) or peripheral visual cues that indicated, where the key would appear (“visual-orienting task”, including new scenes) to orient attention towards the most likely location of the target. For both tasks, valid trials (i.e., target presented at the memorized or the visually-cued location) were compared to neutral trials, which did not include any information about the position of the key. The imaging results revealed a common activation of the fronto-parietal network both for memory-orienting and for visual-orienting. Common clusters of activation included nodes of the dorsal attention network, namely in the intraparietal sulcus (IPS) and the frontal eye fields (FEF), as well as regions belonging to the ventral parietal cortex (VPC, and specifically in the angular gyrus, AG), plus the inferior premotor cortex. In addition, the imaging results revealed that the hippocampus (HPC) and the adjacent parahippocampal cortex activated significantly more in valid vs. neutral trials, selectively for memory- vs. visually-guided spatial orienting. These findings demonstrated that memory-guided attention engages the fronto-parietal networks, traditionally associated with perceptual attention (e.g., see Cabeza et al. 2008; Rosen et al. 2015; see also Sestieri et al. 2017), as well as medial temporal regions typically associated with the processing of scenes, binding of contextual information and retrieval from LTM (Diana et al. 2007; Eichenbaum et al. 2007; Epstein et al. 2003).

The finding that memorized information about objects position in natural scenes can be used to guide spatial attention raises the question of how different frames of reference are combined to re-map the memorized position into the to-be-attended spatial location. Extensive work on scene representation showed that multiple frames of reference are used along both the dorsal occipito-parietal and the ventral occipital-temporal pathways (Byrne et al. 2007). In the posterior parietal cortex and, specifically, in the IPS, egocentric spatial maps are formed transiently (Byrne et al. 2007; Dhindsa et al. 2014). They are then transformed into stable allocentric representations by two ventral regions that selectively encode environmental features: the parahippocampal place area (PPA, Epstein and Kanwisher 1998; Epstein et al. 1999) and the retrosplenial cortex (RSC, Epstein 2008; Epstein et al. 2007a, b; see also Galati et al. 2010). The PPA is involved in the representation of new scene information by encoding scenes as individual snapshots with different views (Park and Chun 2009; Sulpizio et al. 2013). The RSC is thought to support the transformation of egocentric spatial representations, coded in the parietal cortex, into allocentric representations stored in the medial temporal lobe (HPC), and viceversa (Park and Chun 2009; Sulpizio et al. 2013). These complementary functions of the PPA and RSC permit memory retrieval from different viewpoints, via mental self-rotation and reorientation mechanisms (Dhindsa et al. 2014).

In the current framework of memory-guided attention, we, therefore, asked how different frames of reference contribute to visual search, when attention control can make use of memorized spatial information to find targets in natural scenes. Accordingly, first we prompted the participants to learn the position of target-stimuli embedded in real-world scenes (“encoding phase”). Later on, we asked them to search for and discriminate the target-stimuli that were again presented embedded in the same scenes, but now displayed at low visibility/contrast (“retrieval phase”, with high demand of top-down control). Critically, to uncouple the role of the different frames of reference (egocentric/screen-centered vs. allocentric/scene-centered), in some of the trials the scene was presented from a different viewpoint compared with the encoding phase. That is, the position of the target did not change with respect to the scene (e.g., target located on a room’s carpet), but it changed with respect to the viewer/screen (e.g., target in the left visual field at “encoding”, but in the right visual field at “retrieval”).

We hypothesized that memory-guided attention would facilitate target search irrespective of viewpoint change, thus highlighting that (egocentric) visuo-spatial orienting can take into account (allocentric) spatial information about the memorized position of objects in natural scenes (see Exp. 3 in Jiang and Swallow 2014, showing a behavioral advantage when the participants searched for repeated targets at fixed positions within natural scenes, even when the viewpoint changed across trials). Based on the involvement of fronto-parietal regions in attention guidance by memory (e.g., Stokes et al. 2012; Summerfield et al. 2006; see also Rosen et al. 2015), and evidence that the dorsal posterior parietal cortex is involved in perspective transformations during scene processing (Lambrey et al. 2012; Sulpizio et al. 2013, 2016), we expected increased activity in the IPS during memory guided attention, irrespective of the viewpoint change. Our current findings support this hypothesis, demonstrating that the dorsal parietal cortex can not only make use of spatial information stored in LTM, but that it does so by taking into account changes of perspective. The latter appears pivotal for attention control in everyday life, which typically entails continuous changes of viewpoint.

## Materials and methods

### Participants

Twenty-four healthy volunteers participated in the study. Three participants were excluded from data analysis because of within-fMRI-run head-movements larger than 3 mm or 3°, leaving 21 participants for the final analyses (14 females; mean age 27 years; range 20–33 years). All participants were right-handed and had normal or corrected-to-normal (with contact lenses) visual acuity. All of them received an explanation of the procedures and gave written informed consent. The study was approved by the independent Ethics Committee of the IRCCS Fondazione Santa Lucia (Scientific Institute for Research Hospitalization and Health Care).

### Paradigm

**Fig. 1:**
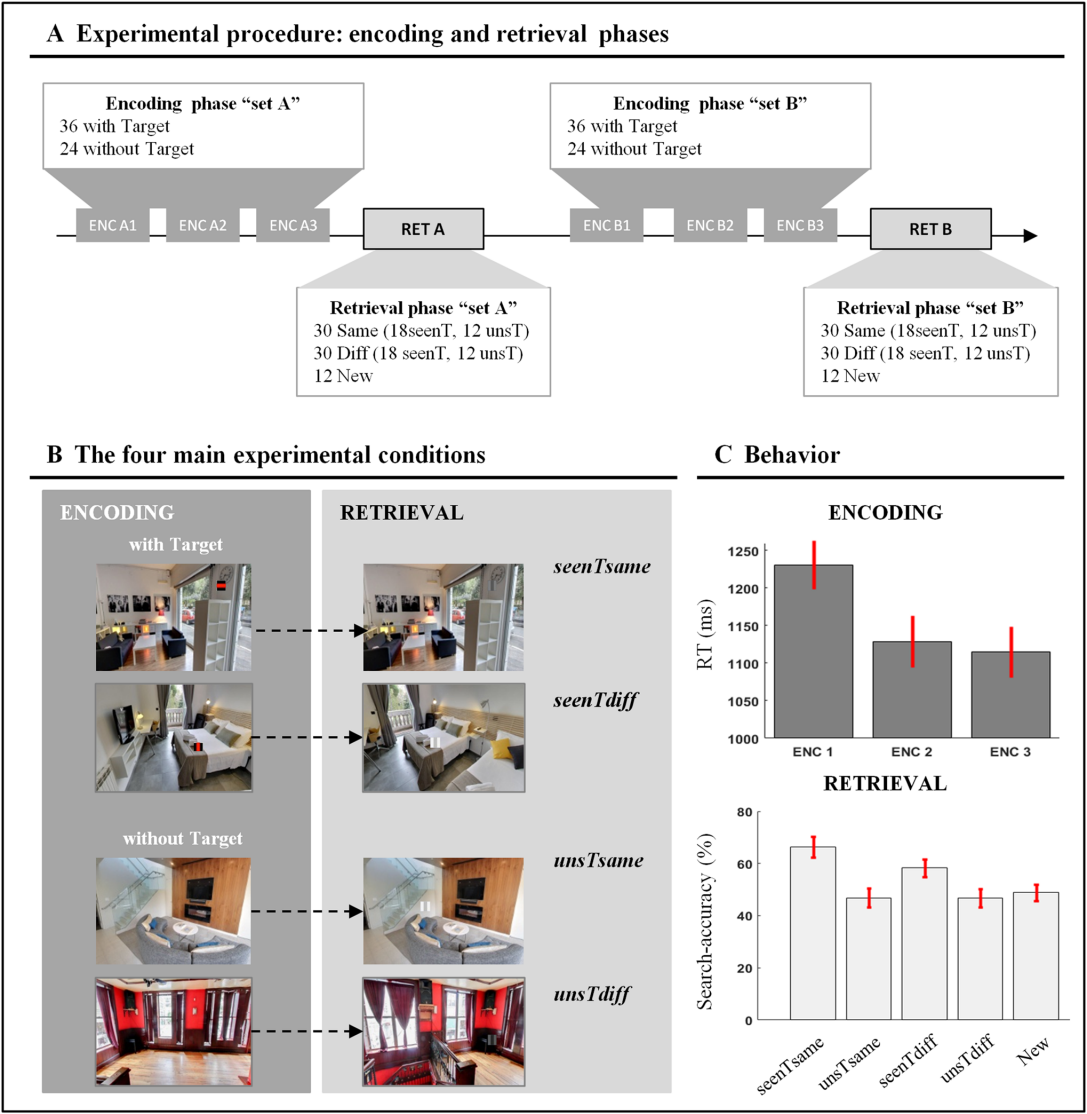
Experimental procedure and behavioural results. **a** Illustration of the experimental procedure. The experiment comprised an encoding phase (ENC), when the participants could memorize the position of highly salient/visible targets superimposed on pictures of natural scenes, followed by a retrieval phase (RET), when the particpants had to search for and discriminate targets presented in the same position as during ENC, but now with low contrast/visibility (see also panel B). Each participant underwent two sequences of ENC and RET, including two different sets of stimuli/pictures. The ENC of each set was repeated 3 times (ENC 1–3), with randomized presentation orders. Each ENC included 36 scenes with the salient target and 24 scenes without any target. During RET all of the scenes included a low contrast target. During both ENC and RET, the task of the participant was to find the target and to indicate the orientation of the bar embedded in the target square. **b** Main experimental conditions. During RET, the participants viewed the scenes that they had previously seen during ENC (*seenTsame, seenTdiff, unsTsame, unsTdiff*) or completely new scenes (*New*, not shown here). The “old” scenes either contained (*seenT* trails) or did not contain (*unsT* trials) the target when initially presented during ENC. Moreover, these “old” scenes could be presented either from the same viewpoint (*same*) or from a different viewpoint (*diff*) compared to ENC. Please note that for display purposes the targets shown here are not to scale (they were enlarged to make them more visible). **c** Reaction times at ENC and search-accuracy at RET. The RTs diminished over the three blocks of ENC (1–3), indicating that the participants learned the position of the target during this phase (note that during encoding the target was highly salient and accuracy was at ceiling, 97%). By contrast, during retrieval the visibility of the target was low (cf. also panel B), and the participants often failed to find the target within the available response window (88% of the error were “omissions”). The analysis of search-accuracy at RET highlighted that participants found the target more often when the scenes included the target during the encoding phase (*seenT* conditions). This effect of attention guidance by memory was present also when the scene-view changed between ENC and RET (cf. *seenTdiff* vs *unsTdiff*; bars 3–4), demonstrating that memory-guided attention can take into account changes of viewpoint. The *New* condition was not significantly different from the *unsT* conditions. The error bars represent the standard error of the mean

The experiment comprised two phases that were performed during fMRI: encoding and retrieval/search phase. At encoding, the participants were asked to discriminate a visual target (a small square with a vertical or horizontal stripe) that was superimposed on pictures of real-world scenes (Fig. 1a). The encoding phase was repeated three times, with the aim of enhancing the likelihood of learning the spatial location of the target within each scene. After a short break, the retrieval phase started. The participants were presented with the same scenes viewed at encoding, but half of them were viewed from a different perspective. The task was again to discriminate the visual targets (vertical/horizontal stripe). The target was always located in the same position with respect to the scene. However, when the scene was shown from a different viewpoint, the position of the target with respect to the participant/screen changed between the encoding and retrieval phase. To prompt the participants using memorized information about the target position, the targets were now presented at a low contrast (Fig. 1b). Additional control conditions included searching for and judging targets in new scenes (not presented at encoding) and in scenes that during the encoding phase did not contain any target.

The analysis of the imaging data considered activity during the retrieval/search phase, testing the hypothesis that stored information about the target position would contribute to search-related activity in the fronto-parietal cortex and, most critically here, that this would occur irrespective of viewpoint changes (cf. contribution of scene/allocentric vs. screen/egocentric space for memory-guided orienting of spatial attention).

### Stimuli and task

The visual stimuli were back-projected on a screen behind the fMRI scanner. Subjects laid in the scanner and viewed the visual display through a mirror mounted on the MRI headcoil (total display size 20 degrees of visual angle, 1024 × 768 screen resolution, 60 Hz refresh rate). Stimulus presentation was controlled with Cogent2000 (www.vislab.ucl.ac.uk/Cogent/) running on MatLab 7.1 (MathWorks, Natick, MA).

Visual stimuli consisted of pictures of real-world indoor scenes, collected through Google Street View. Each scene was taken from three different views: 0° (central view), 60° to the left and 60° to the right of the central view. There were a total of 144 scenes used in the experiment, plus an additional set of 10 scenes used as practice trials to familiarize with the encoding phase. For each participant, the 144 experimental stimuli were divided in two sets of 72 pictures each (“set A” and “set B”), administered consecutively (see Fig. 1a). For each set of pictures, the procedure included three blocks of encoding followed by one block of retrieval/search (see Fig. 1a). For the encoding phase, each of the three blocks included 60 pictures. Each picture was shown for 2.5 s, followed by a variable inter-trial interval ranging from 1.5 to 2.5 s. Thirty-six of the scenes included a to-be-judged target. The target was highly “salient”, i.e., a black square that included a red bar oriented either vertically or horizontally (0.4 × 0.4° of visual angle; Fig. 1b). Participants were instructed to explore the scenes overtly (freely moving their eyes), search for the target and judge whether the red bar was oriented vertically or horizontally. They responded by pressing one of two response buttons on an MRI-compatible button box. Participants were allowed to answer during the presentation of the scene and for 1200 ms after the offset of the image.

In the remaining 24 scenes, no target was presented and the participants did not make any response. Scenes with and without targets were intermixed and presented in an unpredictable order. The participants were explicitly told that, if a target was present, they had to memorize the target position, because this would help them performing the subsequent task (retrieval phase). In the 3 encoding blocks, the participants viewed the same 60 scenes, presented in a different random order. The viewpoint and the target position, in the scenes that included a target, remained the same for all the encoding blocks.

After the three encoding blocks and a delay of approximately five minutes, the participants performed the retrieval/search phase. During this session, the participants viewed 72 indoor scenes (i.e., the 60 scenes viewed at encoding, plus 12 new scenes). All scenes included a to-be-judged target square (0.4 × 0.4° of visual angle) that now was presented at a low contrast. Color and luminance of the target matched the image background at the target location (central bar: + 20% of the average RGB pixel intensities of the target region; two lateral bars: − 20% of the average intensities). As a result, the target visibility was low. Thus, while at encoding stimulus-driven saliency could strongly contribute to guide attention during target search, now these exogenous factors were minimized with the aim of maximizing the contribution of internally-stored information about the target position instead (i.e., memory-guided attention).

Out of the 72 scenes presented in the retrieval phase, 60 were seen during the encoding phase (“old” scenes), while 12 were completely new scenes (“new” scenes: *New* condition). Out of the 60 “old” scenes, 36 included the target at encoding, thus allowing participants to use target-related memory to guide spatial attention at retrieval. Half of these scenes were presented from the same viewpoint across the two phases (“seen target – same view” condition: *seenTsame)*, while for the other half of the scenes, the viewpoint at retrieval was randomly shifted of 60 degrees towards the left or right compared with the encoding phase (“seen target – different view” condition: *seenTdiff*). As a result of this, in all *seenTdiff* trials the target changed visual hemifield between the ENC and RET. In retinal/image coordinates the change of target position ranged between 7.2 and 12.5 visual degrees (see also Epstein et al. 2005, 2007a, b, who used shifts of analogous amplitudes).

The remaining 24 “old” scenes also included the target, but these targets were not displayed during the encoding phase. Again, at retrieval, half of the scenes were presented from the same viewpoint as at encoding (“unseen target—same view” condition: *unsTsame*), while the other half entailed a different viewpoint (“unseen target—different view” condition: *unsTdiff;* see Fig. 1b). These images allowed us to control for any overall effect of scene-memory and of viewpoint change, when assessing specifically the role of memory for the target position and any related influence of the viewpoint-change.

Each picture was presented for 4 s, with a variable inter-trial interval ranging from 3 and 5 s. As for the encoding phase, the participants were instructed to search for the target-square and, if they found the low contrast target, to indicate whether the bar was oriented vertically or horizontally. Subjects were allowed to answer during the presentation of the scene and for 1200 ms after the offset of the image.

### Magnetic resonance imaging

A Siemens Allegra (Siemens Medical Systems, Erlangen, Germany) operating at 3T and equipped for echo-planar imaging (EPI) acquired functional magnetic resonance images. A quadrature volume head coil was used for radio frequency transmission and reception. Head movement was minimized by mild restraint and cushioning. Thirty-two slices of functional MR images were acquired using blood oxygenation level dependent imaging (3 × 3 mm, 2.5 mm thick, 50% distance factor, repetition time (TR) = 2.08 s, time echo (TE) = 30 ms), covering the entirety of the cortex.

### fMRI data analysis

The imaging data were analyzed by using statistical parametric mapping (SPM12; Wellcome Department of Cognitive Neurology) implemented in MATLAB 7.5 (The MathWorks Inc., Natick, MA) for data preprocessing and statistical analyses. Each participant underwent 8 fMRI runs: 6 runs of 140 volumes each, corresponding to the 3 blocks of encoding for each picture set (sets A and B); and 2 runs of 288 volumes each, corresponding to one block of retrieval for each picture set. In line with the aim of the current study, that was to investigate the role of different frames of reference for memory-guided attention, we analyzed and report here the imaging data pertaining to the retrieval/search phase.

The fMRI data underwent a standard pre-processing procedure that included realignment, slice timing, normalization and smoothing. After having discarded the first four volumes of each fMRI-run, rigid-body transformation was used to correct for head movement and slice-acquisition delays were corrected using the middle slice as reference. All images were normalized to the SPM12 template and finally were spatially smoothed using an isotropic Gaussian Kernel of 8 mm full-width at half-maximum (FWHM) to increase the signal-to-noise ratio.

Statistical inference was based on a random effects approach (Penny and Holmes 2004), which comprised two steps: first-level analyses estimating contrasts of interest for each subject followed by second-level analyses for statistical inference at the group level (with non-sphericity correction; Friston et al. 2002).

For each subject, the first-level general linear model (GLM) included 8 conditions. The main conditions of interest were 5 and comprised only correct trials, when the participant found the low-salience target and responded correctly to the bar-orientation task. These were: seen target—same view (*seenTsame*); seen target—different view (*seenTdiff*); unseen target—same view (*unsTsame*); unseen target—different view (*unsTdiff*); new scenes (*New*). In addition, we modeled omissions and wrong responses. The omissions, when the participant did not find the target and did not provide any response in the 5.2 s window, were further divided into two trial-types depending on whether the scene was “old/seen” or “new” (see also below). All wrong responses, when the participant provided a wrong answer to the bar-orientation task, were modeled as a separate trial-type and not considered further in the data analysis. Each trial was modeled as an event time-locked to the onset of the picture, with a duration of 2400 ms, corresponding to the average search time for the correct trials (i.e., the period when the participant made use of memorized information to perform the target search; see below “Results” section). All trials were convolved with the SPM12 hemodynamic response function. The six parameters of head movements resulting from the rigid-body realignment were included as covariates of no interest. The time series at each voxel were high-pass filtered at 220 s and pre-whitened by means of autoregressive model AR(1). Following the estimation of the GLM parameters, we created 4 linear contrasts subtracting the *New* condition from each of the four “old” trial-types, considering only correct trials and averaging across the two fMRI runs of the retrieval phase.

The corresponding 4 contrast-images per participant were entered into a within-subject ANOVA for statistical inference at the group-level. The main aim of the experiment was to test for the effect of attentional guidance by memory and, more specifically, to assess the role of ego- vs. allocentric spatial information for this. Accordingly, we compared trials comprising scenes that at encoding contained the target vs. scenes that were presented without any target (i.e., the main effect of “target present/absent”: *seenT vs. unsT),* and we tested for any modulation of this effect as a function of the view-change (i.e., interaction between “target-present/absent” and “scene-view”: [*seenTsame vs. unsTsame*] vs. [*seenTdiff vs. unsTdiff*]). The statistical threshold was set to p-FWE = 0.05, cluster-level corrected for multiple comparison at the whole brain level (cluster size estimated at a voxel-level threshold of p-uncorrected = 0.001).

For completeness, we also performed a set of additional analyses assessing the effects of task-execution, scene-memory and search-accuracy. For these analyses, we computed 3 specific contrasts at the single-subject level that were then assessed at the group-level using 3 separate one-sample *t* tests. To highlight the whole circuit involved in the current retrieval task (task-execution), we averaged the 4 main trial-types (*seenTsame, seenTdiff, seenTdiff, unsTdiff*). To investigate the effect of scene-memory, we contrasted the 4 main trial-types (all including old/seen scenes) with the *New* condition, considering correct trials only. Finally, we investigated the effect of search-accuracy by contrasting the 4 main trial-types (target found within seen/old scenes) vs. *omissions* considering only trials that included seen/old scenes. It should be noticed that these additional comparisons are confounded with a variety of factors, including target-discrimination and motor-responses (see also “limitations section”, in the Discussion), and they were performed only to relate our main results about memory-guided attention with more general functions of the parietal cortex (cf. Figure 3b, and Discussion section). For the group-level analyses (*t* tests), the statistical threshold was set to p-FWE = 0.05, corrected for multiple comparisons at voxel-level and considering the whole brain as volume of interest.

## Results

### Behavioral data

At encoding, the target was highly visible and indeed the accuracy was high (mean = 97%, range = 92–100%). To assess whether participants learned the target position within each scene, we computed target reaction times separately for each of the three successive presentations. We then performed paired t-tests to highlight the effect of repetition during the ENC phase. This analysis showed that the participants were faster to respond to the targets upon the second and third presentations compared with the first presentation [*T*(20) = 5.82, *p* < 0.001, and *T*(20) = 6.43, *p* < 0.001, respectively], while the difference between the third and second presentation was not statistically significant (*p* = 0.215; see Fig. 1c, top panel). These results indicate that the participants successfully memorized the target position during the encoding phase.

At retrieval, the participants had again to report the orientation of the target-bar, but now this appeared in the scene at a much lower contrast than at encoding (cf. Methods and Fig. 1b). The participants had to search for the target and could use information stored in memory only for scenes that contained the target at encoding (*seenT*-trials). Overall, the number of correct target discriminations was now much lower than during encoding (mean = 50%, range = 24–68%), with the vast majority of errors comprising “no-response” (misses: 88%), indicating that the participant failed to find the target within the allowed response time window (i.e., 5.2 s overall). The latter finding confirms that the manipulation of target-salience/visibility permitted reducing the contribution of any stimulus-driven signal at retrieval, hence encouraging the participants to use internal/memorized information to find the target. Accordingly, for all subsequent analyses (behavior and fMRI) we considered specifically the “search-accuracy”: that is, comparing correct trials (target found and correct bar-discrimination) vs. omissions, when the participant did not find the target and did not provide any response. The few trials when the participant provided an incorrect answer to the bar-discrimination were excluded.

The search-accuracy data were submitted to a within-subjects ANOVA with the factors of “target-presence” at encoding (*seenT* vs. *unsT*) and “scene-view” (*Same* vs. *Diff*) at retrieval with respect to encoding (Fig. 1c, bottom panel). To evaluate the influence of memory on attention guidance, we compared the search-accuracy for old-scenes that contained the target during the encoding phase with the search-accuracy for old-scenes that during the encoding phase were presented without any target (main effect of “target presence”). Furthermore, we tested whether the viewpoint changes between encoding and retrieval modulated this effect of memory on attention (“target-presence” by “scene-view” interaction). The ANOVA revealed a main effect of target-presence [*F*(1, 20) = 31.7, *p* < 0.001], with greater search-accuracy when the participants were presented with scenes that comprised the target during encoding (*seenT*, mean = 58%), than scenes that at encoding were presented without any target (*unsT*, mean = 44%). The ANOVA also indicated statistical trends for the main effects of scene-view [*F*(1, 20) = 3.9, *p* = 0.063; with higher accuracy for trials without any viewpoint-change] and for the interaction between two factors [*F*(1, 20) = 3.6, *p* = 0.071]. Most importantly here, post-hoc comparisons (two-tailed one-sample t-tests, Bonferroni corrected for multiple comparisons: *n* = 6, alpha-level = 0.008) showed that the participants were significantly more accurate for *seenT* vs. *unsT*, both in the *Same* view condition [66% vs. 47%; *t*(20) = 5.3, *p* < 0.001] and in the *Diff* view condition [58% vs. 47%; *t*(20) = 3.6, *p* = 0.002; compare bar 1 vs. bar 2, and bar 3 vs. 4, in Fig. 1c, bottom panel]. The latter finding demonstrates that the participants could use memorized information about the target position to guide target-search during the retrieval phase and, critically, that they could do so even when there was a change of viewpoint between encoding and retrieval/search. It should be stressed that because search-accuracy refers to whether the participant did or did not find the target, these values can potentially drop to 0% and 50% accuracy does not constitute chance-level performance.

For completeness, we also conducted a series of paired *t* test to compare the four main experimental conditions against the baseline condition comprising the *New* scenes (i.e., scenes not seen at encoding). The comparison between the *seenT* trials and the *New* condition revealed significant differences [*t*(20) = 4.3, *p* < 0.001 and *t*(20) = 2.6, *p* = 0.016; for *Same* and *Diff* view, respectively]. By contrast, we did not observe any significant difference between the *unsT* scenes and the *New* condition [*Same*: *t*(20) = − 0.7, *p* = 0.510, and *Diff*: *t*(20) = − 0.8, *p* = 0.439]. These additional results further support the notion that participants could use target-related information stored during the encoding phase (*seenT* trials) to find the target during the retrieval/search phase.

Finally, we also analyzed the participants’ reaction times for the correct trials. A within-subjects ANOVA with the factors of “target-presence” and “scene-view” revealed only a significant interaction between the two factors (*F*(1, 20) = 6.8, *p* = 0.018), with faster reaction times for *seenT* vs. *unsT* trials in the *Same* view condition only (2315 vs. 2541 ms; *t*(20) = 3.6, *p* = 0.002). However, it should be noticed that given the low visibility of the target and the limited response window (5.2 s), here search-accuracy rather than speed best reflected the contribution of memory to visual search.

### fMRI data

#### Main analysis: memory-guided attention

The main fMRI analysis aimed to identify regions involved in attentional guidance by memory and any related influence of view-change. Accordingly, we considered activity during the retrieval/search phase of the task. Also, it should be noticed that this main analysis included only correct trials: that is, trials in which the participant found the low contrast target and reported the correct orientation of the target-bar.

**Fig. 2:**
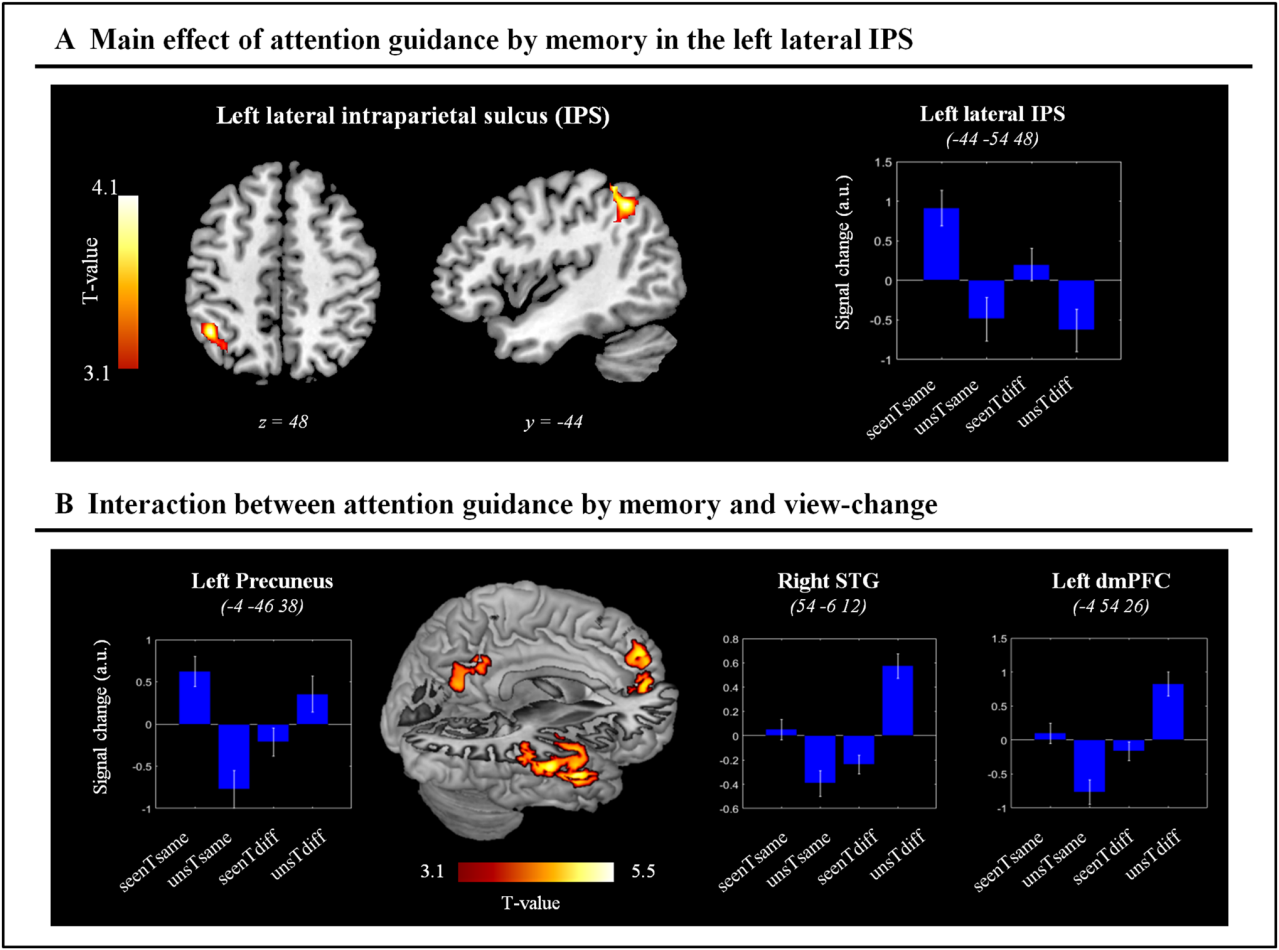
Attention guidance by memory: fMRI activation during the RET phase. **a** Axial and sagittal sections of the main effect of memory-guided attention (“*seenT* > *unsT*”, correct trials only), showing the significant activation of the lateral L IPS. The signal plot indicates that this region was more active when the participants could use memorized information about the target position in the scene, even if the viewpoint changed between ENC and RET. **b** Three-dimensional rendered projection, with cut-out to expose the medial surface of the brain, and signal plots for the three clusters that showed a significant interaction between memory (*seenT* vs. *unT*) and viewpoint (*Same* vs. *Diff*). In two regions (right superior temporal gyrus, STG and the dorsomedial prefrontal cortex, dmPFC), the signal plots indicate that the interaction was driven primarily by the *unsTdiff* condition, suggesting for these regions activation related to processes other than memory of the target position (see Discussion section). By contrast, in the Precuneus the *seenTsame* condition also contributed to the interaction (bar 1 in the leftmost plot). All the signal plots show the mean-adjusted activity (sum = 0) across the four conditions, and therefore, negative values should not be interpreted as de-activations. The signal is expressed in arbitrary units (a.u.) and the error bars represent the standard error of the mean. Clusters of activation are rendered at p-unc. = 0.001, minimum cluster size = 50 voxels

First, we compared trials comprising scenes that during the encoding phase were presented with or without the target (*seenT* vs. *unsT:* main effect of “target presence”). This revealed a significant cluster of activation in the left IPS (L IPS), peaking at the coordinates *x*, *y*, *z* = − 44, − 54, 48. Figure 2a shows the anatomical location of the significant cluster and the related signal plot of the peak voxel. We used citoarchitectonic maps available in SPM12 to assess whether the cluster comprised primarily areas in the lateral or the medial IPS, or other regions of the VPC. This revealed that out of the 201 voxels that could be assigned to a citoarchitectonically defined region, 153 (76%) were located in the lateral portion, hIP1-2, 25 in the medial portion, hIP3 (12%), and only 8 voxels (< 4%) were located in the VPC. Thus, while the peak of the cluster was located relatively ventrally, the bulk of the activation was found in the lateral L IPS. The signal plot highlights that the activity in the lateral L IPS was larger for *seenT* than *unsT* trials, irrespective of the viewpoint, *Same* or *Diff* (Fig. 2a, compare bars 1 & 3 vs. bars 2 & 4).

We sought to confirm these results with two additional analyses. First, at the whole brain level, we tested the effect of “target presence” separately for *Same* and *Diff* conditions. For both contrasts, this revealed peaks of activation in the left IPS (*Same*: *x*, *y*, *z* = − 38, − 62, 42, *t* = 4.24; *Diff*: *x*, *y*, *z* = − 42, − 48, 50, *t* = 3.11). Second, we used MarsBaR (Brett et al. 2002) to analyze the average activity of the main left IPS cluster. This confirmed the presence of a memory effect in both viewing conditions (*Same*: *t* = 3.70; *Diff*: *t* = 1.79) and showed a main effect of view point (*“Same* > *Diff”,* *t* = 1.78), while the interaction was not significant (*t* = 1.33). Please note that these additional tests are not independent from our main whole-brain analysis (Fig. 2a), and therefore, we have not included the corresponding *p* values. Notwithstanding that, these confirmatory tests support that the L IPS was involved in attention guidance by memory also when there was a change of viewpoint between the encoding of the target position and the subsequent search phase.

Next, we tested whether there was any brain region, where the change of viewpoint between encoding and retrieval modulated the activity associated with attention guidance by memory: i.e., the interaction between “target-presence” and “scene-view”. This revealed three clusters of activation located in the right superior temporal gyrus, dorsomedial prefrontal cortex and precuneus (STG, dmPFC and Precuneus; Fig. 2b and Table 1). In the STG and dmPFC, the interaction was driven by higher activity of the *unsTdiff* condition (see bar 4 in the signal plots of these two regions in Fig. 2b), while in the Precuneus, the *seenTsame* condition mainly contributed to this interaction (see bar 1 in the signal plots of this region in Fig. 2b). By contrast, we did not find any region activating specifically during the *seenTdiff* condition.

**Table 1:** Attention guidance by memory

	p-FWE-corr	Cluster size	*X*	*y*	*Z*	*T* value
Main effect of attention guidance by memory						
Lateral L IPS	0.015	245	− 44	− 46	58	4.03
Influence of scene-view change on attention guidance by memory						
R STG	< 0.001	1095	54	− 6	− 12	5.50
L dmPFC	< 0.001	511	− 4	54	26	4.65
L Precuneus	< 0.001	483	− 4	− 46	38	4.04

Anatomical locations, peak coordinates in MNI space (Montreal Neurological Institute), and statistical values for the main effect of memory-guided attention (*seenT* minus *unsT*, irrespective of view-change); and for the interaction between memory and perspective ([*seenTsame*—*unsTsame*]—[*seenTdiff*—*unsTdiff*]). *p* values are corrected for multiple comparisons at the cluster level, considering the whole brain as the volume of interest. Cluster sizes defined at p-unc. = 0.001. *Lateral L IPS* left lateral intraparietal sulcus, *R STG* right superior temporal gyrus, *L dmPFC* left dorsomedial prefrontal cortex, *L Precuneus* left precuneus

#### Additional analyses: task-execution, scene-memory and search-accuracy

**Fig. 3:**
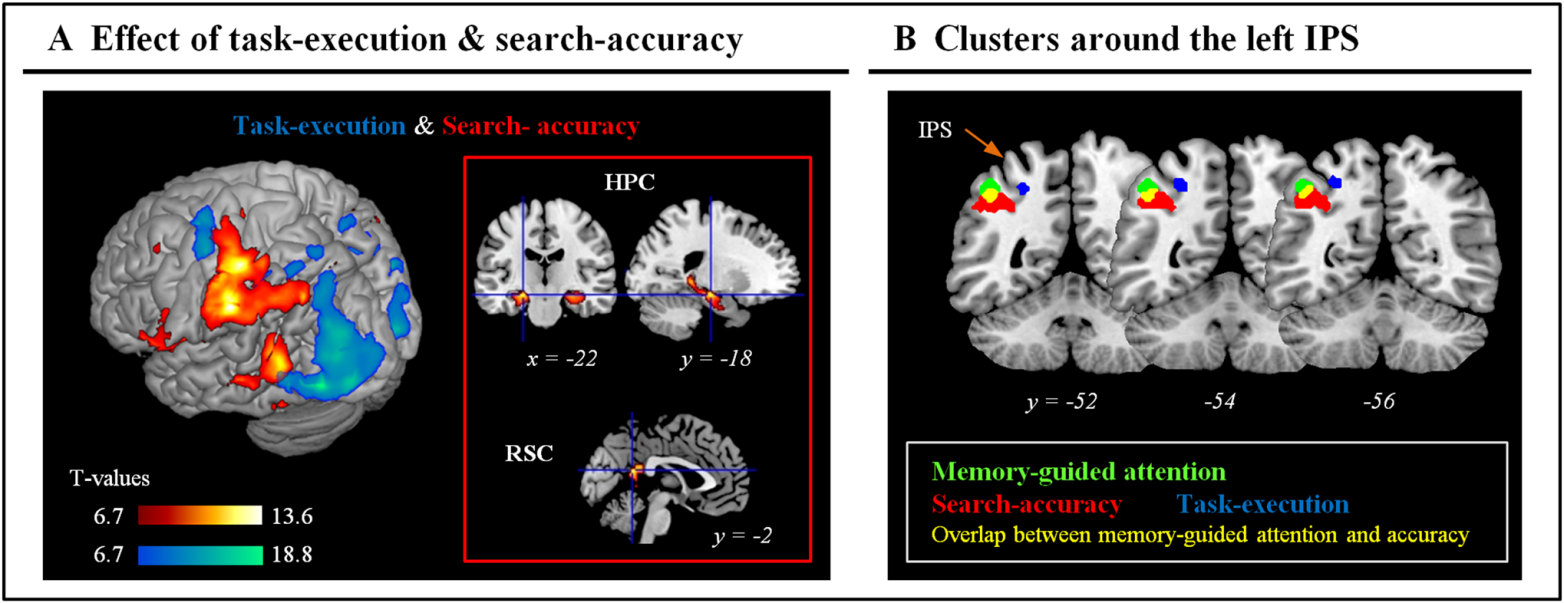
Task execution and search-accuracy. **a** Overall effect of task-execution and search-accuracy. The task-execution contrast averaged activity for the 4 conditions of interest (correct trials only), irrespective of target-memory and view-change. The search-accuracy contrast compared the same 4 conditions against the omissions (i.e., when the participant did not find the target), considering only trails with old/seen scenes. The three-dimensional rendered projections show the two contrasts: task-execution in blue/winter-colours and search-accuracy in red/hot-colours (p-FWE-corr. = 0.05 at the voxel-level). The task-execution contrast highlighted dorsal fronto-parietal activations (including the medial IPS, see also panel B) and the visual occipital cortex (see also Table 1, reporting several others regions not visible in the rendered projection). The search-accuracy contrast revealed activation of the left motor cortex related to the manual responses, but also of the ventral parietal cortex, lateral temporal regions, the hippocampus (HPC) and the retrosplenial cortex (RSC). The clusters of activation within anatomically defined HPC (Neuromorphometrics atlas) and RSC (BA29, in WFU PickAtlas v3.0) are displayed in the inset at p-unc. = 0.001 (see Table 2, for the whole-brain corrected p-values in these regions). **b** Coronal sections showing the anatomical layout of the three activation clusters found around the L IPS: memory-guided attention, in “green” (see also Fig. 2a); task-execution and search-accuracy, in “blue” and “red”, respectively (cf. panel A). These thresholded maps indicate some overlap between memory-guided attention and task-accuracy in the angular gyrus (AG; overlap rendered in yellow), while the task-execution appears engage a distinct region in the medial IPS; see Discussion section. The task-execution and search-accuracy clusters are rendered at p-FWE-corr. = 0.05 at the voxel-level; the effect of memory-guided attention at p-FWE-corr. = 0.05 at the cluster-level (cf. Fig. 2a)

For completeness, we also investigated the full network of areas involved in the current task, irrespective of memory-guided attention. For this, we first examined the overall pattern of activation associated with the execution of the task, averaging activity related to the four main experimental conditions (*seenTsame*, *seenTdiff*, *unsTsame* and *unsTdiff*). This revealed a large set of regions including the visual cortex and dorsal fronto-parietal attention regions (Fig. 3a, in blue; and see Table 2 for the full list of activated areas). In the IPS, the cluster of activation was now found more medially (medial bilateral IPS) as compared to the lateral activation observed for attention guidance by memory (see Fig. 3b, in blue).

**Table 2:** Overall effects of task execution and search-accuracy

	p-FEW-corr.	*x*	*y*	*z*	*T* value
Overall effect of task-execution					
L Fusiform	< 0.001	− 26	− 50	− 10	16.75
R Fusiform	< 0.001	28	− 46	− 12	17.23
L Precuneus	< 0.001	− 16	− 60	10	7.70
R Precuneus	< 0.001	20	− 58	16	11.85
Medial L IPS	< 0.001	− 26	− 54	48	8.43
Medial R IPS	< 0.001	26	− 58	48	7.63
L SPL	< 0.001	− 16	− 62	56	8.46
R SPL	< 0.001	18	− 58	54	8.35
L SPL/SMG	< 0.001	− 34	− 40	42	8.34
L HPC	< 0.001	− 20	− 26	− 4	7.48
R HPC	< 0.001	22	− 26	− 4	7.82
L Precentral	< 0.001	− 38	− 8	52	10.63
R Precentral	< 0.001	46	10	28	8.99
L SMA	< 0.001	− 4	12	46	14.10
R SMA	< 0.001	4	8	54	9.74
L FEF	< 0.001	− 24	0	62	9.52
R FEF	< 0.001	26	2	46	8.97
R IFGOp	< 0.001	46	10	28	8.99
L Insula	< 0.001	− 30	20	− 2	10.27
R Insula	< 0.001	32	24	0	8.42
Overall effect of search-accuracy					
L Fusiform	< 0.001	− 46	− 58	− 22	8.09
L Fusiform	< 0.001	46	− 52	− 24	7.76
L Precuneus	0.001	− 6	− 66	40	7.59
R Precuneus	0.001	6	− 60	42	7.30
L AG	< 0.001	− 40	− 58	42	8.92
R AG	< 0.001	46	− 60	42	8.47
L SMG	< 0.001	− 56	− 32	34	13.64
R SMG	< 0.001	48	− 40	50	9.46
L Postcentral	< 0.001	− 56	− 24	46	10.37
R Postcentral	< 0.001	58	− 20	46	10.10
*L anterior HPC	0.003	− 22	− 18	− 14	8.40
*L posterior HPC	< 0.001	− 16	− 36	2	10.58
*R posterior HPC	0.024	20	− 36	6	7.15
L MTG	< 0.001	− 50	− 60	6	11.28
R MTG	< 0.001	62	− 34	0	10.09
R ITG	< 0.001	54	− 62	− 6	8.72
L Precentral	< 0.001	− 36	− 24	54	9.74
L FEF	0.006	− 12	6	64	7.81
R SFG	0.006	14	52	32	7.60
L MFG	< 0.001	− 34	24	38	7.40
R MFG	0.001	28	20	52	8.29
R IFGOp	0.001	58	14	6	7.85
R IFGTr	< 0.001	50	42	0	7.30
L Insula	< 0.001	− 40	0	12	11.56
R Insula	< 0.001	42	0	4	9.77
L mPrecentral	< 0.001	− 6	− 24	46	9.36
L mCgG	< 0.001	− 2	8	40	10.28
L aCgG	< 0.001	− 6	36	20	7.97
R pCgG	< 0.001	2	− 28	36	7.91
*RSC	0.013	0	− 50	18	7.55
R Thalamus proper	0.002	10	− 26	10	7.87
L Putamen	< 0.001	− 16	6	− 12	8.26
L Cerebellum exterior	< 0.001	− 34	− 48	− 28	8.62
R Cerebellum exterior	< 0.001	26	− 52	− 24	10.85
R Cerebellar vermal lobules I-V	< 0.001	2	− 46	− 4	8.36

Anatomical locations, peak coordinates in MNI space (Montreal Neurological Institute), and statistical values for the general effects of task execution and search-accuracy. *p* values are corrected for multiple comparisons at the voxel level considering the whole brain as the volume of interest. The overall effect of task execution considered all trials types (vs. Rest), irrespective of conditions; the overall effect of search-accuracy compared correct trials vs. incorrect trials, irrespective of target-presence and scene-view conditions. *L/R* left/right hemisphere, *aCgG* anterior cingulate gyrus, *Cerebellum Exterior* cerebellum exterior, *Cerebellar Vermal Lobules I-V* cerebellar vermal lobules I-V, *FEF* frontal eye field, *Fusiform* fusiform gyrus, *HPC* hippocampus, *IFGOp* inferior frontal gyrus, opercular part, *IFGTr* inferior frontal gyrus, triangular part, *Insula* insula; *(medial)* *IPS* intraparietal sulcus, *ITG* inferior temporal gyrus, *mCgG* middle cingulate gyrus, *MFG* middle frontal gyrus, *mPrecentral* precentral gyrus, medial segment, *MTG* middle temporal gyrus, *pCgG* posterior cingulate gyrus, *Postcentral* postcentral gyrus, *Precentral* precentral gyrus, *(dorsal) Premotor* premotor cortex, *RSC* retrosplenial cortex, *SFG* superior frontal gyrus, *SMA* supplementary motor area, *SMG* supramarginal gyrus, *Thalamus Proper* thalamus proper. (*) For HPC and RSC, we report whole-brain corrected peaks located within anatomically defined areas (HPC: Neuromorphometrics atlas, in SPM; RSC: BA29, in WFU PickAtlas v3.0)

We sought to further relate the effect of memory-guided attention to other processes ought to engage in the current experiment by testing for the effects of scene-memory and search-accuracy. We assessed scene-memory by comparing the 4 main conditions that included scenes presented during the encoding phase ("old" scenes) vs. the *New* scenes, but this did not reveal any significant effect after correction for multiple comparisons (see Discussion section). Next, we tested for areas involved in the accurate performance of the search task by comparing the 4 main experimental conditions vs. omission-trials, considering only trials with old/seen scenes. This revealed a large set of areas (Table 2 and Fig. 3a, in red), including motor regions associated with the manual response, but also activation of the VPC bilaterally, as well as the retrosplenial cortex (RSC) and the hippocampus (HPC; see Fig. 3a, inset-frame on the right), two well-known regions involved in scene processing and coordinate transformations (Byrne et al. 2007; see also “Discussion”). In the left hemisphere, the posterior part of the VPC cluster overlapped with the most inferior/lateral part of the cluster associated with memory-guided attention (see Fig. 3b, overlap displayed in yellow). Because such cluster overlap is fully dependent on the thresholds of the whole-brain statistical maps, we sought to confirm the effect of search-accuracy in the left lateral IPS associated with memory-guided attention using MarsBaR (Brett et al. 2002). In agreement with the overlap observed in the whole-brain analyses, this revealed a strong effect of search-accuracy in the left lateral IPS (*t* = 8.80).

We investigated further the possible relationship between the left IPS and regions related to scene processing and coordinate transformations (i.e., HPC, RSC and Precuneus; Byrne et al. 2007) using an explorative analysis of functional connectivity. For the left IPS and the Precuneus we considered voxels that activated for the main effect of memory (Fig. 2a) and the memory-by-view interaction (Fig. 2b), while anatomical masks were used for the HPC (Neuromorphometrics atlas, in SPM) and for the RSC (BA29, in WFU PickAtlas v3.0; Maldjian et al. 2003, 2004). For each region we computed average blood-oxygen dependent (BOLD)-time series. These were then high-pass filtered (cut-off = 128 s) and, for each subject, pair-wise correlation coefficients between the 4 regions were computed and Fisher-transformed. For statistical inference at the group level, the transformed-coefficients were submitted to two-tailed one-sample t-tests considering a Bonferroni corrected alpha-level = 0.008 (*n* = 6). The results showed a significant functional coupling between all pairs of areas (all ps < 0.001). This exploratory analysis suggests that the left IPS and was functionally coupled with other regions involved in scene memory and coordinate transformation during the current task.

In sum, the imaging data highlighted that the lateral L IPS plays a central role in mediating the effect of memorized information about the target position during search in natural scenes. This effect of memory on visuo-spatial attention was found irrespective of view changes between the initial encoding of the target position and the successive target search. The latter finding supports the hypothesis that the parietal cortex can make use of allocentric/scene-centered spatial information, when memory contributes to guide visuo-spatial attention (cf. also behavioral results).

## Discussion

The previous literature established that memory can contribute to guide visuo-spatial attention, as evidenced during search for objects placed in previously learned locations (Rosen et al. 2015; Summerfield et al. 2006). Here we investigated for the first time the role of different frames of reference (egocentric vs. allocentric) during memory-guided visual search in natural scenes. We designed our study so as to maximise the role of memory by using low-contrast target-stimuli that reduced any effect of stimulus-driven signals during search. Crucially, across trials, we manipulated whether the subjective viewpoint of the scene was fixed or it changed between the encoding and the retrieval/search phase. This allowed us to test the hypothesis that memory-guided search can make use of allocentric spatial information (i.e., the position of the target with respect to the scene), when there is a change of the target position with respect to the participant's subjective viewpoint (i.e., the egocentric frame of reference).

### Main results: changes of viewpoint in memory-guided attention

Behaviourally, we found evidence that the participants made use of memorised spatial information about the target position to find the low-contrast targets. The participants were able to find the targets more often when they had already encountered the target during encoding, as compared to when they had no information about the target location (i.e., when they had seen the scene, but without the target during the encoding phase). Furthermore, the participants were faster and more accurate when at retrieval they were presented with scenes from the same viewpoint as during the encoding, than when they were presented with scenes from a different perspective (interaction between memory and viewpoint, with a statistical trend for the accuracy data). This fits with the previous results showing that viewpoint changes result in a reduction of behavioral performance, consistently with the existence of viewpoint-dependent memory representations (Burgess 2006; Schmidt et al. 2007; Sulpizio et al. 2016). However, most relevant here, we found that participants were significantly more accurate to find seen vs. not-seen targets, both when the scene viewpoint was fixed and when it changed (cf. Figure 1c). This demonstrates that memory-guided attention can take into account viewpoint-independent information about the target stimulus location in natural scenes.

At a neuroimaging level, we found that the L IPS activated when the participants could use memorised information to successfully find low-contrast targets (Fig. 2a). The engagement of the parietal cortex is consistent with previous imaging work on memory-guided attention (Cabeza et al. 2008, 2011; Rosen et al. 2015; Sestieri et al. 2010; Summerfield et al. 2006). Most importantly, here we demonstrated guidance-related activity in the L IPS irrespective of perspective changes between the encoding and the subsequent retrieval/search. The latter demonstrates that the L IPS contribution to memory-guided attention can take into account scene-centred, allocentric spatial information.

The finding that memory-guided attention can operate using position-signals across different reference frames (egocentric/allocentric) is in good agreement with the Byrne, Becker, and Burgess’s (BBB) model of spatial memory (Byrne et al. 2007). This model proposes the existence of dynamic interactions between egocentric representations in the Precuneus and allocentric representations in the medial temporal cortex during the encoding and the retrieval of spatial scenes. Long-term spatial memories would be stored in allocentric coordinates in the HPC and surrounding regions, and transformed to egocentric representations via the RSC and the posterior parietal cortex. While the BBB model mainly addresses these processes in the framework of navigation, planning and imagery, related retrieval and transformation processes are likely to take place here. Specifically, the participants may use specific landmarks in the scene (e.g., objects embedded in the scenes) as cues to reconstruct the scene layout and guide (egocentric) visual search towards the (allocentric) stored position of the target stimulus, when the latter information is available (i.e., in *seenT* trials; see also Sulpizio et al. 2013, for possible distinctions between object-based vs. environment-based transformations). Previous imaging results supporting the BBB model came, for example, from a study by Dhindsa and colleagues (2014). The participants were asked to imagine a configuration of objects from a different perspective compared with the previously learned viewpoint, thus requiring the transformation between egocentric and allocentric representations. The results showed that the brain regions involved in the reference-frame transformation matched those predicted by BBB model and included the IPS. In line with the BBB model, our current findings show that the IPS can make use of allocentric information when searching for a visual target, whose position is fixed with respect of the external, scene-based frame of reference.

Together with the viewpoint-independent memory effect in L IPS, our data also highlighted the involvement of the Precuneus, which activated during memory-guided attention with unchanged viewpoint (Fig. 2b), as well as the HPC and the RSC that activated when the participants successfully found the target irrespective of memory and view (see Fig. 3a). Explorative analyses of functional connectivity revealed correlated activity in the left IPS, the Precuneus, the HPC and the RSC, suggesting that these may belong to a common network and jointly participate to the current search task. The RSC is well-known to be part of the “core” network activated by scene stimuli compared with other visual categories (Walther et al. 2009; Hodgetts et al. 2016). The RSC primarily contains spatial layout information, rather than information about objects (Harel et al. 2013). Furthermore, Hodgetts et al. (2016) recently demonstrated preferential scene responses in both the HPC and the “core” scene-processing network, suggesting that the HPC can be also considered part of the network, given its role in high-order scene perception (Lee et al. 2012). The current task involved multiple relevant processes, including retrieving scene layout details and the target-position (in *seenT* condition), performing coordinate transformations, detecting and discriminating the target, as well as controlling strategically the allocation of visuo-spatial attention. All these processes were tightly coupled in time, which makes it difficult to tease apart the relative role of each region in the different processes (see also *Limitations* section, below).

One possible prediction could be that regions involved in coordinate transformations would activate specifically when the trial required retrieving the target-position following a change of perspective (*seenTdiff* condition). These trials should generate greater demands on the relevant coordinate transformation circuit, including the RSC and/or the parahippocampus (e.g., Epstein 2008; Epstein et al. 2017; Park and Chun 2009; see Galati et al. 2010, for a review; and Bicanski and Burgess 2018, for a recent computational model). However, our fMRI analyses did not reveal any region with this specific pattern of activation. The interaction between memory and viewpoint change revealed activations in the Precuneus, STG and dmPFC. The Precuneus responded preferentially when the perspective did not change (*seenTsame),* possibly reflecting the relevance of egocentric reference frames in this region (Byrne et al. 2007; and see above). By contrast, in the right STG and the left dmPFC activity increased for the *unsTdiff* condition: that is, when there was a change of viewpoint and the participants did not have any prior information about the target position (cf. Figure 2b). Arguably, this was the most challenging search condition for the participants. Activation of the superior temporal cortex has been linked to many attention-related processes, including shifts attention and eye-movements (Corbetta et al. 1998) and global vs. local processing (Fink et al. 1996). Most recently, Rosen and colleagues (2016) compared stimulus- vs. memory-guided search in natural scenes (see also below) and found larger activation for stimulus- than memory-guided attention in the lateral temporal cortex. Accordingly, we suggest that here the activation of the STG reflects a greater requirement of stimulus-related attention, rather than any specific effect linked with the change of perspective in the *unsTdiff* condition. The role of the dmPFC may be linked with self-projection during LTM retrieval (Cabeza and St. Jacques 2007). Using a naturalistic protocol involving wearable cameras at encoding and subsequent scene retrieval under fMRI, St. Jacques and colleagues (2011) showed activation of the dmPFC when participants were presented with scenes depicting familiar places, but from a perspective that they had not perceived themselves (i.e., images recorded by a different participant). Despite their task did not involve any visual search as here, one may speculate that some general self-referential process related to the retrieval of known scenes but from a different prospective may account for the dmPFC activation in both studies.

### Additional findings: attention and memory in the parietal cortex

Beside any issue specific to the change of perspective, our results in the L IPS also contribute to the debate about the interplay between attention and memory in the parietal cortex. While addressing this issue was not the aim of our study/design, the results of our additional analyses about the overall effects of task-execution and search-accuracy provide us with potentially relevant information. Some authors posited that mechanisms of attention control and memory retrieval rely on analogous selection processes (“Attention to Memory”, AtoM, model, Cabeza et al. 2008; Ciaramelli et al. 2008). The AtoM model proposes that the dorsal parietal cortex (DPC, that includes the IPS) allocates attention during memory retrieval according to the subject’s goals (cf., top-down attention control in perception; Corbetta and Shulman 2002) while modulating at the same time the activity in the medial temporal lobe. By contrast, the VPC (comprising the AG and SMG) would operate as a “circuit breaker” to capture attention towards relevant memories / retrieval outputs (cf., bottom-up attention; Corbetta and Shulman 2002). In support of this hypothesis, Cabeza and colleagues (2011) reported overlapping activation in DPC (including the IPS) for top-down search guided either by memory or perceptual signals, while the VPC activated for target-detection, again both in the memory and the perceptual domains. However, other authors argued against this view. For example, Sestieri and colleagues (2010) showed that searching for information stored in memory (i.e., details about events in a movie) engaged the lateral portion of the IPS and the AG, while looking for a target object while watching the video (i.e., stimulus-related attention) activated the medial and posterior parts of the IPS. Differences in task and stimulus material may explain these inconsistencies, together with the fact that the distribution of spatial attention was not controlled/accounted for in these studies. Given the key role of the IPS in visuo-spatial coding, such control appears pivotal when addressing the interplay between memory and attention.

A series of recent studies by Rosen and colleagues (2015, 2016, 2018) directly investigated spatial aspects of memory-guided attention (see also the pioneering study of Summerfield et al. 2006). The first two studies (Rosen et al. 2015, 2016) made use of a change detection task, entailing voluntary search-related processes analogous to those engaged in the current study that made use of low-salience targets. Specifically, Rosen et al. (2015) investigated spatio-topic maps in the IPS, while the participants directed attention towards different positions of natural scenes, either based on stimulus-driven attention (exogenous cues, superimposed on the scene), or while using memorized information about the position of the target/change. The results showed largely overlapping effects for the two tasks along the whole IPS, consistent with common spatial effects for perceptual and memory-guided search (cf. Cabeza et al. 2008). Nonetheless, this study did not compare directly the two orienting conditions. In a subsequent study using the same change-detection paradigm (Rosen et al. 2016), the authors replicated the common effects of attention and memory both in the lateral and the superior/posterior IPS, but also showed that while the former engaged primarily during search from memory, the latter activated mostly during the perceptual task, in line with the findings of Sestieri et al. (2010). The key role of the lateral IPS for memory-guided spatial attention was confirmed in a subsequent study, using simpler displays with arrays of isolated objects (Rosen et al. 2018). The experiment now included a condition with endogenous central cues (rather than exogenous cues), as well as an additional control task that entailed memory-retrieval, but without any spatial component. The results confirmed the role of the lateral IPS that was found to activate also when memory-guided spatial attention was compared with the non-spatial retrieval control task. The study also replicated the activation of the posterior Precuneus and the posterior callosal sulcus that Rosen and colleagues (2018) suggested constituting a functionally-connected network for memory-guided attention (see also Rosen et al. 2015; and note that also in the current study we found correlated activity between the left IPS and the Precuneus, cf. the exploratory analyses of functional connectivity).

Here, we used a paradigm in which both the encoding and retrieval tasks emphasised the (non-verbal) spatial dimension and minimized any influence of (perceptual) exogenous signalling in all conditions (cf. low-salience targets). Using cytoarchitectonic probabilistic maps (Eickhoff et al. 2005), we found that most of the activated voxels were located in hIP1 and hIP2 in the lateral bank of the IPS, while only 12% were in the medial bank (hIP3) and only 8 voxels (< 4%) extended to the VPC. This is consistent with the view that memory-guided attention engages primarily the lateral IPS (Rosen et al. 2015, 2016; Sestieri et al. 2010). Importantly, it should be noticed that unlike all the studies discussed above, here the activation of the lateral IPS was found by comparing conditions when the participants performed the same task: that is, they always searched for, and successfully found, low-salience targets embedded in scenes that they had seen before. The only difference was whether the scenes included or not the target during the encoding phase. This rules out any possible influence of sensory- or task-related processes, which may have contributed to the effects reported in the previous studies that compared memory-guided conditions with perceptual conditions involving exogenous cues (Rosen et al. 2015; Summerfield et al. 2006), endogenous cues (Rosen et al. 2018) , or used low-level baseline conditions (rest, Cabeza et al. 2011; passive “no-task” control, in Rosen et al. 2016).

The current protocol was not designed to compare stimulus- vs. memory-guided search, but the results of the different contrasts related to task-execution, search-accuracy and memory-guided attention suggest some segregation between lateral and medial aspects of the IPS (see Fig. 3b). The memory-related cluster in the lateral IPS partially overlapped with the effect of search-accuracy that was found to activate the VPC (see Fig. 3a, in red). The search-accuracy effect in the VPC included both the SMG, which most likely reflects the detection of the behaviorally-relevant target (Corbetta and Shulman 2002; see also Cabeza et al. 2011), as well as the AG. The role of the AG in memory retrieval has been studied extensively and it is often associated with the representation of information that has been retrieved with high-levels of confidence and/or rich episodic details (e.g., Wheeler and Buckner 2004; see also Gilmore et al. 2015; Sestieri et al. 2017; Wagner et al. 2005, for theoretical perspectives). Here, the activation of the AG reflected the outcome of the attention search task, irrespective of prior knowledge about the target position, making unlikely any interpretation based solely on memory retrieval and possibly hinting at some segregation between retrieval and post-retrieval processes in the lateral IPS and AG, respectively (Hutchinson et al. 2014; Sestieri et al. 2017).

The activation of these lateral regions could be set apart from task-execution that was found to engage the medial bank of the IPS instead (see Fig. 3b, in blue). The task-execution contrast highlighted also activation of dorsal premotor cortex/FEF, as well the supplementary motor area, visual cortex and the insulae, which corresponds to the typical pattern of activation observed in (perceptual) visual search tasks (Corbetta et al. 1995; Fairhall et al. 2009; Nobre et al. 2003; and see Ogawa and Macaluso 2015, for a search task using natural scenes). The anatomical separation of the clusters in the lateral vs. medial IPS should be interpreted with some caution, as it depends on the statistical thresholds, but—overall—the picture emerging here aligns with proposals emphasizing some distinction between (perceptual) attention control in dorsal fronto-parietal regions, which would include the medial IPS, and regions mediating the impact of memory on such control, which would rely on the lateral IPS.

## Limitations and future work

The current study follows up the results initially reported by Summerfield and colleagues (2006), showing that LTM can guide attention during visual search within naturalistic scenes. We extended their findings examining how perspective changes between encoding and retrieval may affect memory-guided search. This required the inclusion of a relatively high number of conditions (memory *x* view-change, plus new scenes) that, once we also factored-in the behavioral performance (cf. omission trials), resulted in a relatively low number of trial-repetitions per condition (e.g., on average 10 correct trials per subject, for each of the two *unsT* conditions). Nonetheless, there are several points that should be considered. First, and most important, our main contrasts compared approx. 30 vs. 20 trials (main effect of “seen targets”) and 25 vs. 25 trials (interaction between “target-presence and view-change”). Second, it should be noticed that here we included relatively long “events”, with a duration of 2.4 s. These events are expected to generate a larger BOLD response compared to short events (e.g., 500 ms events, in Huettel and McCarthy 2001; who recommended 20–30 trials for event-related designs).

A second limitation that we should acknowledge concerns our additional analyses. These tested for the overall effect of task-execution and search-accuracy comparing conditions that differed for many different processes. The task-execution contrast considered the average activity of the 4 conditions of interest and the resulting activations included a mixture of sensory, motor and task-related processes. The search-accuracy contrast compared correct trials with omissions and revealed not only regions involved in successful search, but also activations related to target discrimination and response (cf. activation of motor areas). Despite these caveats, these comparisons provided us with some additional information about the relationship between the memory-guided effect in the L IPS and the more general involvement of the parietal cortex in memory and attention control (cf. the previous section). These findings should be interpreted with caution, because the amount of overlap between the different contrasts depends on the chosen thresholds. Future studies may address these relationships in a more direct manner using dedicated localizer tasks to identify areas activated by specific memory and/or attention processes and investigating the effect of perspective changes during memory-guided search within these independently-defined functional regions.

Along the same line, future studies should also consider separating some of the processes characterizing our four main conditions. On the one hand, some parametric manipulation of the angle of view-change may enable revealing more specifically brain activity associated with coordinates transformation. On the other hand, a combination of task instructions (e.g., “judge scene”, “search target”) and some temporal separation between scene- vs. target-presentation should permit isolating activity related to scene retrieval vs. target detection, in both cases with or without view-changes. Moreover, the temporal separation of these processes would facilitate testing models of effective connectivity addressing the dynamic interactions between IPS, HPC, RSC and Precuneus. This would contribute to elucidating the mechanisms underlying coordinates transformation during memory-guided search.

## Conclusions

In sum, the present study shows that the lateral L IPS can use scene-centred spatial memory during visual search in natural scenes. We suggest that the lateral L IPS feeds the dorsal attention network (including the medial IPS and premotor regions) with prior knowledge about the target position in the scene. The relevant scene-centred information may be stored in the medial temporal cortex and, at the time of search, transformed to viewer-centred coordinates via a network comprising the RSC, the Precuneus and the IPS (Byrne et al. 2007). Future work should seek to disentangle the role of these different regions by experimentally uncoupling processes related to target-/scene-retrieval, coordinates transformation and visual search (attention shifting and target detection). We conclude that the lateral L IPS is a central hub for memory-guided attention and that its role is likely to be important in everyday life, when—most typically—the subjective viewpoint will change between the encoding and the retrieval of objects position in the real world.

## Data Availability

The datasets generated during and/or analysed during the current study are available from the corresponding author on reasonable request.

## References

[CR1] Bicanski A, Burgess N (2018). A neural-level model of spatial memory and imagery. eLife.

[CR3] Brett M, Anton J-L, Valabregue R, Poline J-B (2002) Region of interest analysis using an SPM toolbox. Presented at the 8th International Conference on Functional Mapping of the Human Brain, Sendai, Japan. NeuroImage, 16(2): 497

[CR4] Burgess N (2006). Spatial memory: how egocentric and allocentric combine. Trends Cogn Sci.

[CR5] Byrne P, Becker S, Burgess N (2007). Remembering the past and imagining the future: a neural model of spatial memory and imagery. Psychol Rev.

[CR6] Cabeza R, St. Jacques JP (2007). Functional neuroimaging of autobiographical memory. Trends Cogn Sci.

[CR7] Cabeza R, Ciaramelli E, Olson IR, Moscovitch M (2008). The parietal cortex and episodic memory: an attentional account. Nat Rev Neurosci.

[CR8] Cabeza R, Mazuz YS, Stokes J (2011). Overlapping parietal activity in memory and perception: evidence for the attention to memory model. J Cogn Neurosci.

[CR9] Chun MM, Jiang Y (1998). Contextual cueing: implicit learning and memory of visual context guides spatial attention. Cogn Psychol.

[CR10] Chun MM, Turk-Browne NB (2007). Interactions between attention and memory. Curr Opin Neurobiol.

[CR11] Ciaramelli E, Grady CL, Moscovitch M (2008). Top-down and bottom-up attention to memory: a hypothesis (AtoM) on the role of the posterior parietal cortex in memory retrieval. Neuropsychologia.

[CR12] Corbetta M, Shulman GL (2002). Control of goal-directed and stimulus-driven attention in the brain. Nat Rev Neurosci.

[CR13] Corbetta M, Shulman GL, Miezin FM, Petersen SE (1995). Superior parietal cortex activation during spatial attention shifts and visual feature conjunction. Science.

[CR14] Corbetta M, Akbudak E, Conturo TE (1998). A common network of functional areas for attention and eye movements. Neuron.

[CR16] Dhindsa K, Drobinin V, King J (2014). Examining the role of the temporo-parietal network in memory, imagery, and viewpoint transformations. Front Human Neurosci.

[CR17] Diana RA, Yonelinas AP, Ranganath C (2007). Imaging recollection and familiarity in the medial temporal lobe: a three-component model. Trends Cogn Sci.

[CR18] Eichenbaum H, Yonelinas A, Ranganath C (2007). The medial temporal lobe and recognition memory. Annu Rev Neurosci.

[CR19] Eickhoff SB, Stephan KE, Mohlberg H (2005). A new SPM toolbox for combining probabilistic cytoarchitectonic maps and functional imaging data. NeuroImage.

[CR20] Epstein RA (2008). Parahippocampal and retrosplenial contributions to human spatial navigation. Trends Cogn Sci.

[CR22] Epstein R, Kanwisher N (1998). A cortical representation of the local visual environment. Nature.

[CR23] Epstein R, Harris A, Stanley D, Kanwisher N (1999). The parahippocampal place area: recognition, navigation, or encoding?. Neuron.

[CR24] Epstein R, Graham KS, Downing PE (2003). Viewpoint-specific scene representations in human parahippocampal cortex. Neuron.

[CR25] Epstein RA, Higgins JS, Thompson-Schill SL (2005). Learning places from views: variation in scene processing as a function of experience and navigational ability. J Cogn Neurosci.

[CR26] Epstein RA, Higgins JS, Jablonski K, Feiler AM (2007). Visual scene processing in familiar and unfamiliar environments. J Neurophysiol.

[CR27] Epstein RA, Parker WE, Feiler AM (2007). Where am i now? Distinct roles for parahippocampal and retrosplenial cortices in place recognition. J Neurosci.

[CR28] Epstein RA, Patai EZ, Julian JB, Spiers HJ (2017). The cognitive map in humans: spatial navigation and beyond. Nat Neurosci.

[CR29] Fairhall SL, Indovina I, Driver J, Macaluso E (2009). The brain network underlying serial visual search: comparing overt and covert spatial orienting, for activations and for effective connectivity. Cereb Cortex.

[CR30] Fink GR, Halligan PW, Marshall JC (1996). Where in the brain does visual attention select the forest and the trees?. Nature.

[CR31] Friston K, Penny W, Phillips C (2002). Classical and Bayesian inference in neuroimaging: theory. NeuroImage.

[CR32] Galati G, Pelle G, Berthoz A, Committeri G (2010). Multiple reference frames used by the human brain for spatial perception and memory. Exp Brain Res.

[CR33] Gilmore AW, Nelson SM, Mcdermott KB (2015). A parietal memory network revealed by multiple MRI methods. Trends Cogn Sci.

[CR34] Gottlieb J, Hayhoe M, Hikosaka O, Rangel A (2014). Attention, reward, and information seeking. J Neurosci.

[CR35] Harel A, Kravitz DJ, Baker CI (2013). Deconstructing visual scenes in cortex: gradients of object and spatial layout information. Cereb Cortex.

[CR37] Hodgetts CJ, Shine JP, Lawrence AD, Downing PE, Graham KS (2016). Evidencing a place for the hippocampus within the core scene processing network. Hum Brain Mapp.

[CR38] Hollingworth A (2004). Constructing visual representations of natural scenes: the roles of short- and long-term visual memory. J Exp Psychol Hum Percept Perform.

[CR39] Hollingworth A, Williams CC, Henderson JM (2001). To see and remember: Visually specific information is retained in memory from previously attended objects in natural scenes. Psychon Bull Rev.

[CR40] Huettel SA, McCarthy G (2001). The effects of single-trial averaging upon the spatial extent of fMRI activation. NeuroReport.

[CR41] Hutchinson JB, Turk-Browne NB (2012). Memory-guided attention: control from multiple memory systems. Trends Cogn Sci.

[CR42] Hutchinson JB, Uncapher MR, Weiner KS (2014). Functional heterogeneity in posterior parietal cortex across attention and episodic memory retrieval. Cereb Cortex.

[CR43] Jiang YV, Swallow KM (2014). Changing viewer perspectives reveals constraints to implicit visual statistical learning. J Vis.

[CR44] Lambrey S, Doeller C, Berthoz A, Burgess N (2012). Imagining being somewhere else: neural basis of changing perspective in space. Cereb Cortex.

[CR45] Lee AC, Yeung LK, Barense MD (2012). The hippocampus and visual perception. Front Hum Neurosci.

[CR46] Maldjian JA, Laurienti PJ, Burdette JB, Kraft RA (2003). An automated method for neuroanatomic and cytoarchitectonic atlas-based interrogation of fMRI data sets. Neuroimage.

[CR47] Maldjian JA, Laurienti PJ, Burdette JH (2004). Precentral gyrus discrepancy in electronic versions of the talairach atlas. Neuroimage.

[CR48] Maunsell JH (2004). Neuronal representations of cognitive state: reward or attention?. Trends Cogn Sci.

[CR51] Nobre A, Coull J, Walsh V, Frith C (2003). Brain activations during visual search: contributions of search efficiency versus feature binding. Neuroimage.

[CR52] Ogawa A, Macaluso E (2015). Orienting of visuo-spatial attention in complex 3D space: search and detection. Hum Brain Mapp.

[CR53] Olivers CN (2011). Long-term visual associations affect attentional guidance. Acta Physiol (Oxf).

[CR54] Park S, Chun MM (2009). Different roles of the parahippocampal place area (PPA) and retrosplenial cortex (RSC) in panoramic scene perception. Neuroimage.

[CR55] Patai EZ, Doallo S, Nobre AC (2012). Long-term memories bias sensitivity and target selection in complex scenes. J Cogn Neurosci.

[CR56] Penny W, Holmes A (2004) Random-effects analysis. In: Human brain function: second edition 843–850. 10.1016/b978-012264841-0/50044-5

[CR57] Posner MI (1980). Orienting of attention. Q J Exp Psychol.

[CR58] Preston AR, Eichenbaum H (2013). Interplay of hippocampus and prefrontal cortex in memory. Curr Biol.

[CR59] Rosen ML, Stern CE, Michalka SW (2015). Influences of long-term memory-guided attention and stimulus-guided attention on visuospatial representations within human intraparietal sulcus. J Neurosci.

[CR60] Rosen ML, Stern CE, Michalka SW (2016). Cognitive control network contributions to memory-guided visual attention. Cereb Cortex.

[CR61] Rosen ML, Stern CE, Devaney KJ, Somers DC (2018). Cortical and subcortical contributions to long-term memory-guided visuospatial attention. Cereb Cortex.

[CR62] Schmidt D, Krause B, Weiss P (2007). Visuospatial working memory and changes of the point of view in 3D space. Neuroimage.

[CR63] Sestieri C, Shulman GL, Corbetta M (2010). Attention to memory and the environment: functional specialization and dynamic competition in human posterior parietal cortex. J Neurosci.

[CR64] Sestieri C, Shulman GL, Corbetta M (2017). The contribution of the human posterior parietal cortex to episodic memory. Nat Rev Neurosci.

[CR65] St. Jacques PL, Conway MA, Lowder MW, Cabeza R (2011). Watching my mind unfold versus yours: An fMRI study using a novel camera technology to examine neural differences in self-projection of self versus other perspectives. J Cogn Neurosci.

[CR66] Stokes MG, Atherton K, Patai EZ, Nobre AC (2012). Long-term memory prepares neural activity for perception. Proc Natl Acad Sci.

[CR67] Sulpizio V, Committeri G, Lambrey S (2013). Selective role of lingual/parahippocampal gyrus and retrosplenial complex in spatial memory across viewpoint changes relative to the environmental reference frame. Behav Brain Res.

[CR68] Sulpizio V, Committeri G, Lambrey S (2016). Role of the human retrosplenial cortex/parieto-occipital sulcus in perspective priming. Neuroimage.

[CR69] Summerfield JJ, Lepsien J, Gitelman DR (2006). Orienting attention based on long-term memory experience. Neuron.

[CR70] Summerfield JJ, Rao A, Garside N, Nobre AC (2011). Biasing perception by spatial long-term memory. J Neurosci.

[CR71] Wagner AD, Shannon BJ, Kahn I, Buckner RL (2005). Parietal lobe contributions to episodic memory retrieval. Trends Cogn Sci.

[CR72] Walther DB, Caddigan E, Fei-Fei L, Beck DM (2009). Natural scene categories revealed in distributed patterns of activity in the human brain. J Neurosci.

[CR73] Wheeler ME, Buckner RL (2004). Functional-anatomic correlates of remembering and knowing. Neuroimage.

[CR74] Wolfe JM, Horowitz TS (2017). Five factors that guide attention in visual search. Nat Human Behav.

